# Stent-assisted coiling of broad-necked intracranial aneurysms with a new braided microstent (Accero): procedural results and long-term follow-up

**DOI:** 10.1038/s41598-019-57102-6

**Published:** 2020-01-15

**Authors:** Oliver Beuing, Anja Lenz, Aneta Donitza, Mathias Becker, Steffen Serowy, Martin Skalej

**Affiliations:** Department of Neuroradiology, University Hospital Magdeburg, Leipziger Str. 44, Magdeburg, Germany

**Keywords:** Outcomes research, Neurovascular disorders

## Abstract

Intracranial stents have expanded endovascular therapy options for intracranial aneurysms. The braided Accero stent is available for clinical use since May 2015. To date, no clinical reports on the stent are available. Purpose of this study was the evaluation of the safety and efficacy of the Accero stent in stent-assisted coiling. All patients, in whom implantation of the stent was performed, were included. Primary endpoints were good clinical outcome (mRS ≤ 2) and aneurysm occlusion grades 1 and 2 (Raymond Roy Occlusion Classification). Secondary endpoints were procedural and device-related complications with permanent disability or death, complications in the course, and the recanalization rate. Between September 2015 and August 2018, thirty-four aneurysms were treated with stent-assisted coiling using the Accero. Sixteen aneurysms were untreated, four of these were ruptured. Mild neurological complications occurred in 2/34 (5.9%) treatments. Two stent occlusions occurred during follow-up. No patient had a poor procedure- or device-related outcome. After an average of 15 months of follow-up, 28/30 aneurysms were completely or near-completely occluded. The Accero stent proved to be safe and effective in the treatment of broad-based intracranial aneurysms. The complication rate and the rate of successful aneurysm occlusions are similar to those of other stents.

## Introduction

Stent-assisted coiling (SAC) is a long-established therapy option for intracranial aneurysms^1,2^. With this method it is possible to endovascularly treat even complex and broad-necked aneurysms, as stents form a scaffold and thus prevent protrusion or dislocation of coils into the parent artery. Over the years, a variety of stents with different properties have been developed. These include laser-cut stents in open-cell or closed-cell design, as well as braided stents^3–5^. The requirements for all stents are the same: advancement in the catheter and deployment must be easy, they must provide sufficient coverage of the aneurysm neck and be flexible enough to maintain their shape and ensure complete wall apposition even in tortuous vascular anatomy. But above all, usage of the device must be safe and lead to good clinical results with regard to aneurysm occlusion. We present the first results of SAC with a new, braided stent (Accero, Acandis, Pforzheim, Germany).

## Materials and Methods

The data was extracted from a database of our institution that was created for all neurovascular interventions within the framework of a quality control. From this database, the prospectively collected data on all patients, in whom an Accero stent was implanted or intended to be implanted, were extracted and retrospectively analyzed. STROBE guidelines were applied^6^. The study was conducted according to the guidelines and with the approval of the Local Ethics Committee of the University of Magdeburg in compliance with national legislation and the Code of Ethical Principles for Medical Research Involving Human Subjects of the World Medical Association (Declaration of Helsinki). The Local Review Board stated that the retrospective use of the clinical and imaging data does not require separate informed consent.

### Device information

**Figure 1:**
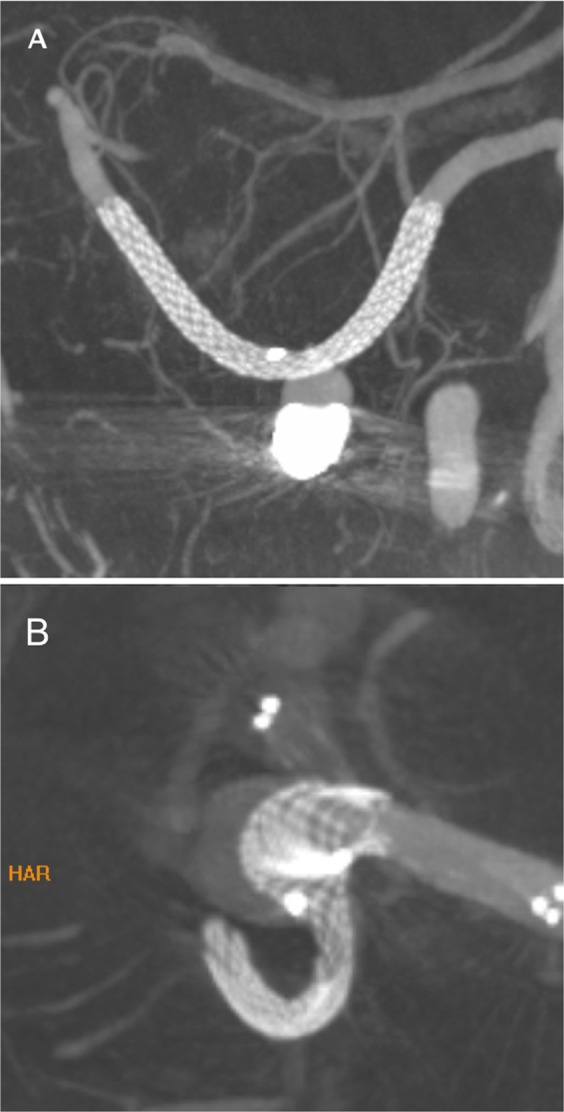
High-resolution volume-of-interest (VOI) imaging of two implanted Accero stents. (**A**) Thick-slab maximum intensity projection after multiplanar reconstruction after deployment of the stent in the left PICA. The stent has opened completely and shows a complete wall-apposition. The metal artifacts caused by the previous coiling do not affect the assessability of the stent lumen. (**B**) Incomplete opening at the distal end of the stent (white arrow). Note the intended bulging of the stent into the aneurysmal lumen (white arrowhead), which, however, substantially increased by trying to reach the distal end of the stent with a balloon catheter. However, the bulging was not sufficient to cover the ostium sufficiently for coiling, so that a second stent was implanted.

The Accero stent is a commercially available, self-expandable braided stent that is approved for the treatment of intracranial aneurysms. The device is characterized by an entire contour visibility due to platinum-nitinol composite wires (Fig. 1). Moreover, the Accero Stent is provided with the BlueXide surface finishing, that aims to optimize hemocompatibility and facilitates stent delivery by using a corrosion-protective surface, ensuring an extremely low nickel ion release^7^. Furthermore, high oxygen and nitrogen intensities of the protective titanium oxide/oxynitride film will work to reduce platelet adhesion. Available diameters range from 2.5 mm to 4.5 mm with lengths up to 25 mm. The Accero stent can be delivered through 0.0165–0.0170” microcatheters. Re-sheathing is still possible when 95% of its length are released.

### Endovascular procedure

All interventions were performed on a biplane angiography system (Artis Q, Siemens Healthineers, Forchheim, Germany) under general anesthesia by one of four neuroradiologists with many years of experience in neurovascular interventions. Written informed consent was obtained prior to the treatment in elective cases. Since the device is approved for stent-assisted coiling, only general information about the risks of stent-assisted coiling with braided stents was provided. The need for informed consent was waived in emergency cases, in such cases, the therapy strategy was determined on an interdisciplinary basis. Commercially available 6F or 8F femoral sheaths and guide catheters were used. In the case of elective treatments, patients were given 100 mg acetylsalicylic acid (ASA) and 75 mg clopidogrel daily for at least 5 days prior to the intervention, the efficacy of both was verified by light emission aggregometry and the Multiplate Analyzer (Roche Diagnostics, Rotkreuz, Switzerland). In addition, heparin adapted to weight was administered intravenously after groin puncture. In patients with subarachnoid hemorrhage, prior to stenting, 500 mg ASA were given intravenously and 180 mg ticagrelor via a nasogastric tube. Post-interventionally, ticagrelor was changed to clopidogrel and efficacy was determined as soon as possible. Clopidogrel was discontinued after 3 months, whereas ASA was given permanently.

The size of the stents was determined by measurements in the DSA working projections, for the choice of length also the local vascular anatomy was considered using 3D-rotational angiography (3D-RA). For the deployment of the stent, a neuroslider 17 (Acandis GmbH, Pforzheim, Germany) was used, which can be used for coiling as well. If Y-stenting was necessary, the second stent was laser-cut (Acclino flex, Acandis GmbH, Pforzheim, Germany) in all cases, which was placed through the meshes of the Accero. High-resolution volume-of-interest (VOI) imaging was used to assess wall apposition and opening at the ends in untreated aneurysms and in those, in which artifacts due to coils didn’t affect the assessment of the stent lumen. At the discretion of the interventionalist, incidental aneurysms were coiled in the same or a second session.

All relevant details of the intervention regarding the aneurysms (size, localization, neck width, aspect ratio, dome-to-neck ratio, prior treatment), the stents (number, size, deployment behavior, advancement or wall adherence), the coiling (jailing, crossing, staged, coils used) and the complications were documented and analyzed.

### Follow-Up

Patients were examined for neurological or other symptoms before and immediately after the intervention as well as at discharge. In addition, the patients were evaluated clinically prior to each follow-up imaging.

According to the imaging follow-up protocol of our institution, a DSA is performed 6 and 18 months after stent implantation, usually in combination with a contrast-enhanced MR-angiography (CE-MRA). After that, only CE-MRA will be performed. In case of abnormal findings (for example suspected reperfusion, intimal hyperplasia or in-stent stenosis), the interval is shortened or re-treatment is performed.

Treatment results for the aneurysms were assessed immediately after the intervention and in the follow-up examinations using the Raymond-Roy occlusion classification (RROC)^8^.

### End-points

Primary end-points were the rate of patients with a good clinical outcome (mRS ≤ 2) and the proportion of patients with complete/near complete aneurysm occlusion (Raymond Roy Occlusion Grades (RROG) 1 and 2, respectively) at the time of latest follow-up. Secondary end-points comprised of complications that were related either to the procedure or the device and caused disabling stroke/death immediately or during the course. Procedure-related complications included thromboembolic events, vasospasm requiring treatment, dissection and perforation, whereas for example detachment or deployment problems, incomplete opening and kinking were rated device-related.

### Statistical analysis

Data were analyzed using IBM SPSS Statistics, Version 24 (Armonk, New York, USA). The two-sided T-test was applied to search for statistically significant differences between subgroups with respect to morphologic parameters. Statistical significance of differences in categorical data between the pretreated and the untreated subgroup was analyzed by a Fisher´s exact test with 2-sided *p*-values.

## Results

Between September 2015 and August 2018, 32 patients with 34 aneurysms received an Accero stent as part of a stent-assisted coiling (SAC). The mean age was 57,1 ± 13,1 years, and twenty-seven (84,4%) patients were female. Four (12,5%) patients had acute SAH with WFNS grades of 1 (n = 3) and 4 (n = 1), respectively. One aneurysm was symptomatic due to mass effect. Approximately half of the patients were retreated after coiling (n = 16) or clipping (n = 2). All aneurysms had a neck width of ≥4 mm and/or a dome-to-neck ratio of ≤2. Twenty-five aneurysms were smaller than 5 mm. For details of the aneurysm characteristics see Table 1. Aneurysm localizations were as follows: anterior communicating artery (n = 13), MCA-bifurcation (n = 7), basilar artery (n = 4), posterior communicating artery (n = 3), pericallosal artery (n = 2), posterior inferior cerebellar artery (PICA, n = 2), internal carotid artery (ICA, n = 1), carotid-t (ICA-T, n = 1) and posterior cerebral artery (n = 1).

**Table 1:** Morphological aneurysm characteristics.

		All (n = 34)	Pretreated (n = 18)	Untreated (n = 16)
Size^a^ (mm)	Mean/SD	5.1 (±4.8)	3.3* (±1.2)	7.0* (±6.4)
	Median	3, 9	3.1	5.2
	Range	1.6–28.7	1.6–*5.7*	2.6–28.7
Neck width (mm)	Mean/SD	4.0 (±1.4)	3.6* (±1.2)	4.6* (±1.6)
	Median	4.0	3.6	4.3
	Range	1.8–7.1	1.8–6.0	2.1–7.1
Max. width^b^ (mm)	Mean/SD	6.2 (±4.6)	4.7* (±1.8)	8.0* (±6.1)
	Median	4.7	4.4	6.8
	Range	1.8–27.4	1.8–8.0	2.7–27.4
Height^c^ (mm)	Mean/SD	5.0 (±4.8)	3.4* (±1.2)	6.9* (±6.4)
	Median	3.9	3.1	5.2
	Range	1.6–28.7	1.6–5.7	2.6–28.7
Aspect Ratio^d^	Mean/SD	1.21 (±0.67)	1.02 (±0.46)	1.42 (± 0.81)
	Median	1.06	0.99	1.19
	Range	0.42–4.03	0.42–2.01	0.70–4.03
Dome-to-Neck Ratio	Mean/SD	1.47 (±0.56)	1.31 (±0.32)	1.64 (±0.71)
	Median	1.33	1.26	1.43
	Range	0.72–3.85	0.72–1.80	0.97–3.85
Vessel Diameter proximal (mm)	Mean/SD	2.7 (±0.60)	2.6 (±0.60)	2.8 (±0.63)
	Median	2, 6	2, 5	2.8
	Range	1.8–4.0	1.8–4.0	1.9–4.0
Vessel Diameter distal (mm)	Mean/SD	2.2 (±0.51)	2.2 (±0.50)	2.1 (±0.51)
	Median	2.1	2.1	2.0
	Range	1.6–3.7	1.7–3.5	1.6–3.7

^a^Measured along the axis from the center of the neck plane to the farthest point on the aneurysm dome^31^. ^b^Maximum transverse diameter perpendicular to the long axis. ^c^Maximum perpendicular distance from dome to neck plane. Calculated by dividing height by neck width. *Significant (p < 0.05) difference between the untreated and pretreated group. As expected, the untreated aneurysms were significantly larger than the reperfused ones. However, aspect ratios (AR) and dome-to-neck ratios (DNR) as well as the vessel diameters didn’t differ significantly.

### Procedural results

SAC with a single Accero stent was planned in 25 aneurysms, and in 9 cases, primary Y-stenting was considered necessary. Deployment of the stent was successful in all 34 treatments. In one case (2,9%), proximal dislocation of the stent occurred by entangling of the pusher wire with the stent struts, which required placement of a second stent. No further rescue stents were required in this or any other patient. So in total, 35 Accero stents were placed in 34 aneurysms. Twenty-eight (82,4%) aneurysms were coiled in the same session, 24 (85,7%) of these with the jailing technique. Two of the patients with SAH had SAC with a single stent, two had Y-Stenting, all without new neurological deficits during the hospital stay.

Complete aneurysm occlusion (RROG 1) was achieved in 18 (52,9%) aneurysms, a neck remnant (RROG 2) was present in 10 (29,4%) aneurysms. Residual filling of the sac was observed in six (17,6%) aneurysms, including two patients, in whom the aneurysm couldn’t be entered for coiling after stenting, in both of which coiling of an anterior communicating artery aneurysm was tried in a second session 8 weeks after stenting. In one of these cases a distinct endothelial layer formed over that portion of the stent that covered the ostium, which prevented probing through the stent meshes. Since the aneurysm was significantly smaller after the stenting alone, it was decided to carry out follow-up examinations. In the second case, the angle between the stent axis and the aneurysm was unfavorable. The attending physician decided to terminate the intervention, although the aneurysm could have been entered from the contralateral side. The patient refused, as the size of the remaining aneurysm was also regredient. In only three of nine patients with Y-Stenting RROG 1 was achieved.

In 4 (11.8%) aneurysms, the distal end of the stent didn’t open completely. In two of these cases, this was not detected under fluoroscopy, but only by VOI imaging, which was performed after deployment. In one case, the stent was unintentionally deployed in an attempt to resheath it. This stent was released until just before the point of no return. In the fourth case, the incomplete opening was accepted, as the interventionalist felt that correction by balloon dilatation would be possible without much effort. However, in no case could the incomplete stent opening be corrected because the balloons could not be advanced to the distal end of the stent. After the 4th issue, VOI imaging was performed before final release in cases where the distal stent opening could not be clearly assessed by fluoroscopy.

Two procedure-related clinical complications occurred. One patient with Y-stenting for an MCA bifurcation aneurysm had multiple embolisms in the ACA- and MCA-territories with mild hemiparesis. She was one of the patients in whom the stent did not open completely distally.

Another patient with an Acom-aneurysm was coiled in a second session eight weeks after uncomplicated single stenting. During coiling a thrombus formed at the site where the stent didn’t cover the ostium. Despite instant medical treatment the patient developed a mild weakness of the foot. Both complications were not disabling (mRS 1 and 2 at discharge, respectively). No other procedural complications were observed.

### Follow-Up

Follow-up examinations were performed in 30 (88.2%) aneurysms, with a mean duration of 444 (range 90–880) days. Reasons for missing follow-up were: death unrelated to the intervention (n = 2, accident and mesenteric infarction due to newly diagnosed atrial fibrillation, respectively), transfer to home country (n = 1) and refusal (n = 1).

**Figure 2:**
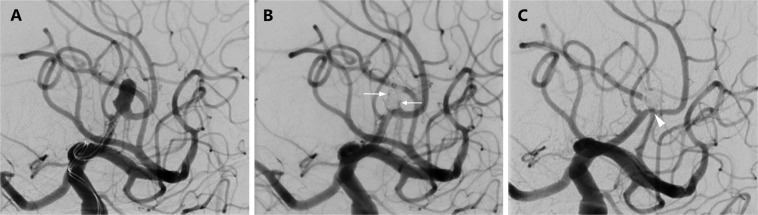
Initial treatment result and follow-up after SAC of an aneurysm at the pericallosal artery with the jailing technique. (**A**) Working projection with microcatheters immediately proximal to the aneurysm. The aneurysm develops from the apex of a sharp curve. A little further distally again sharp bend of the vessel. (**B**) Result immediately after the intervention. Still little inflow into the aneurysm sac (RROG 3, white arrows). Note the slightly different vascular anatomy after stenting. (**C**) In the control-DSA after 6 months complete aneurysm occlusion (RROG 1). Note the discreet intimal hyperplasia in the region of the aneurysm ostium (white arrowhead).

In 21 (70,0%) patients, the aneurysm was completely occluded at the time of the last control. Seven patients improved from RROG 2 or 3 to complete occlusion (see Fig. 2). In two previously occluded aneurysms, a neck remnant developed during the course, and two aneurysms showed progressive filling (RROG 2 to 3), one of which needed re-treatment. Both aneurysms that could not be probed improved from RROG 3 to RROG 1 and 2, respectively. In total 93,3% of the aneurysms showed a complete/near complete occlusion (see Table 2). No change of the stent position was observed. Also, the configuration of the four stents that were distally incompletely open was unchanged.

**Table 2:** Initial and follow-up treatment results.

n = 30		Follow-Up Result		
		RR1	RR2	RR3
**Initial Result**	RR1	14	2	0
	RR2	4	3	2
	RR3	3	2	0
Sum:		21 (70.0%)	7 (23.3%)	2 (6.7%)

Table 1: Comparison of occlusion rates immediately after treatment (initial results) with those after the last imaging (follow-up results) of the whole patient population. The numbers below the main diagonal correspond to improvements. There were no significant differences between untreated and pretreated patients (p = 0.472).

In one patient (3.3%), dual platelet inhibition was discontinued at day 29 after Y-stenting in an outside hospital due to pseudoaneurysm surgery at the puncture site, but without consulting the attending neurointerventionalist. This resulted in an acute occlusion of the Accero stent in the right inferior M2-branch. It was the same patient, who experienced embolism during the intervention. The new event again caused a slight hemiparesis (mRS 2 at discharge). The symptoms improved over time, but didn’t resolve completely. In another patient, asymptomatic occlusion of the A2-segment following SAC of an Acom-aneurysm was detected in the first follow-up DSA. In this patient, the stent also hadn’t opened completely distally. Mild intimal hyperplasia occurred in 6 (20,0%) patients, three of them had Y-Stenting. No in-stent-stenosis was observed. In the patient who had mild foot palsy after coiling, the symptoms completely receded.

## Discussion

The introduction of specially designed self-expandable stents for intracranial use has significantly expanded the spectrum of endovascular aneurysm therapy. The first report on such a stent, the Neuroform, was published in 2002, others followed shortly thereafter^1,9,10^.

A variety of stents with different properties have been developed since then, all with different advantages and disadvantages. Open-cell stents have the disadvantage that the cell size increases significantly at the outer curvature of tortuous vessels, which can lead to coil prolapse^11–13^. In addition, re-sheathing is not possible, so it frequently has to be removed in case of dislocation during deployment^14,15^. On the other hand, laser-cut stents with closed-cell design can ovalize or even kink in tight curves^16^. Migration has also been observed^17^.

Braided stents such as the Accero, which was investigated in this study, should be superior to the laser-cut stents in these regards. They are more flexible and adapt well to the vascular anatomy without kinking. Indeed, no intervention in our series had to be terminated because of technical complications, in all cases an Accero could be placed as intended. In one case, a second stent had to deployed due to detachment problems, though. Also, the wall apposition was complete even in tight curves, no flattening or kinking was observed. However, the stent didn’t open completely distally in 4 cases (11,8%). Incomplete opening not only at the ends has also been observed with other stents, though less frequently as in this study. Usually this can be corrected and in most cases doesn’t cause any clinical symptoms^18–20^. However, one of these patients developed persistent clinical symptoms, but whether these resulted from the incomplete opening is questionable. The embolisms that occurred intra-procedurally did not only affect the vascular territory downstream of the stent, and the stent occlusion after 4 weeks in the same patient happened after discontinuation of the platelet inhibitors. But as another patient with an incomplete opening had an asymptomatic occlusion, at least the later complication must be considered stent-related. In both of these patients, the difference between vessel diameters proximal and distal was each greater than 1 millimeter, and in all four cases the distal vessel diameters were approximately 1.5 millimeters, which is smaller than in most other patients. It may be possible that a small vessel diameter favors the incomplete stent opening. However, by performing a VOI imaging before final release whenever the complete deployment of the stent could not be clearly assessed, this technical complication could be avoided after the fourth case. Overall, the rate of technical difficulties is similar to the 6–11% that are reported for other braided stents^20–22^. With 5, 9% minor neurological intra-procedural complications, one of them with complete remission, and no device- or procedure-related disabling strokes or deaths our results are in line with those of other groups, which also reported no or only low rates of serious complications, regardless of the stent used^19,23–25^.

The Accero stent has a high metal coverage, it is specified by the manufacturer for a stent with the size of 3.5 × 20 mm with 15–19%, depending on the actual diameter. It is thus significantly larger than that of laser-cut stents and comparable to that given for other braided stents^24,26^.

This better coverage of the aneurysm ostium may also result in a greater change in the intra-aneurysmal flow that favors the aneurysmal occlusion in the course. At least in this study, the Raymond Roy occlusion grade improved in nine of the thirteen initially incompletely occluded aneurysms that were controlled, seven of which were completely occluded. Only in two cases (6,7%), there was a recanalization to the aneurysm sac, one required re-treatment. A positive effect of braided stents on the flow was noted by other authors as well^13,27^. On the other hand, better metal coverage might make probing of the aneurysm after stenting more difficult, in particular, when the stent is compressed over the ostium to enhance the effect on hemodynamics. In this study, this occurred in two cases. Although both aneurysms shrank in the course, with the use of braided stents, primarily a one-time procedure in jailing technique should be chosen in order to avoid the possibility that the stents can’t be crossed anymore and thus not be embolized properly.

No coil prolapse was observed in this study. This may also be explained by the high density of the stent struts and better retainment of coils. Thus, braided stents could reduce the complications that arises from coiling in the SAC.

Delayed complications after SAC may arise from interactions of the vessel wall with the implant. In this study, mild intimal hyperplasia was found in six patients (20.0%). This rate is higher than in most other publications already mentioned. However, the changes were only discreet and didn’t require any therapy. In addition, there have been no in-stent stenoses, whereas in the literature a frequency of up to 10% is reported^28^.

However, a therapy should not only be safe, but also efficient. In this study, SAC with the Accero stent resulted in complete/near complete occlusion in 93.3% of the cases, which is in line with the literature. Although an even higher rate of complete occlusion would be desirable, re-treatment of the mostly very small neck remnants would place patients at a disproportionate risk, given the very low risk of rupture of such remnants, especially of unruptured aneurysms^29,30^.

### Limitations

The first limitation is that this study is a retrospective analysis. However, the data were collected prospectively, so that they are qualitatively better than in a purely retrospective survey. Second, this is a single center study with a relatively small number of cases. However, the main purpose of the study was to test the safety and efficacy of the Accero stent and in this regard doesn’t stand out from many other studies that reported the experience with a new device. The neuroradiologists who have implanted the Accero stent are all experienced in neurointerventions and especially in stenting for many years. It can therefore be assumed that a higher number of cases would not have produced a significantly different result in terms of complication and occlusion rates. To test the performance of the Accero over other available stents, however, requires a different study design with more patients. Finally, not all patients had a follow-up examination. Although this can hardly be avoided in the context of patient studies, the results can, of course, be influenced by this. In the vast majority of patients who had a follow-up examination, however, stable or better findings were observed.

## Conclusion

Stent-assisted coiling with the new braided Accero stent appears to be safe and effective. The complication rates are low and the occlusion rates are comparable to those of other stents. Together with its compatibility with a 0.0165 “catheter regardless of the stent diameter, it is a good alternative for the treatment of intracranial aneurysms.

## Data Availability

Any additional unpublished data simply available upon request from the corresponding author.
